# Polarization and US foreign policy: key debates and new findings

**DOI:** 10.1057/s41311-022-00381-0

**Published:** 2022-03-11

**Authors:** Gordon M. Friedrichs, Jordan Tama

**Affiliations:** 1 University of Freiburg, Freiburg, Germany; 2 American University, Washington D.C., USA

**Keywords:** Polarization, US foreign policy, Public opinion, Congress, Presidency, International order

## Abstract

Polarization in the USA has been on the rise for several decades. In this context, few observers expect politics today to stop “at the water’s edge,” as the old cliché goes. But key questions about the relationship between polarization and US foreign policy remain to be fully answered. To what extent are American ideas about foreign policy now polarized along partisan lines? How is polarization changing the foreign policy behavior of the US Congress and President? And how is polarization altering the effectiveness of US foreign policy and influencing America’s role in the world? In this introductory article to our special issue “Domestic Polarization and US Foreign Policy: Ideas, Institutions, and Policy Implications,” we provide an overview of key debates and existing knowledge about these questions, highlight important new findings from the contributions to the special issue, and suggest avenues for further research on this increasingly important topic.

Polarization has become a dominant phenomenon in contemporary American politics (McCarty et al. 2016; McCarty 2019). Partisan division, both among the public and political elites, has consumed American democracy, transforming a political system dependent on compromise into one suffused by hostility, gridlock, and dysfunctional democratic governance (Binder 2015; Lee 2015). Partisans often view supporters of the other party as essentially different, unpatriotic, and morally wrong, which has fostered acts of mutual discrimination, divergent understandings of facts, justifications for antidemocratic behavior, and even outright hate (Finkel et al. 2020). These trends have only become more pronounced with the rise of populism and growing threats of violence against elected officials (Howell and Moe 2020; Itkowitz and DeBonis 2021). 

The term polarization is routinely used in a variety of ways, underscoring the importance of defining the term clearly and distinguishing among types or dimensions of polarization. Broadly, polarization refers to a state in which the opinions, feelings, behaviors, or interests of a group or society become more bimodal and the two modes move further apart.^1^ This broad concept can be broken down into more specific types of polarization. Ideological or preference polarization refers to the polarization of people’s views about public issues, either across-the-board or in particular policy areas (Noel 2013; Carmines and D’Amico 2015; McCarty et al. 2016; Kertzer et al. 2021). Affective polarization, or negative partisanship, refers to sharpening feelings of animosity between people of different political persuasions (Abramowitz and Webster 2016; Mason 2018; Iyengar et al., 2019). Some work also points to the importance of residential or social polarization, characterized by people becoming increasingly separated into communities or groups that interact with each other less (Nall 2015; Alduncin et al. 2017). When any type of polarization overlaps with party identities, partisan polarization exists. Partisan polarization can also be fueled by partisan warfare—the no-holds-barred approach in which politicians seek above all to expand the power of their own party and weaken the other party (Lee 2009; Jacobson 2013; Theriault 2013).

Scholars of American politics have studied the causes and consequences of polarization (Theriault 2008; Persily 2015; Campbell 2016; Thurber and Yoshinaka, 2015; Klein 2020). Various studies suggest that polarization leads to political gridlock and obstructionism, a decline in policy innovation and progress, and even a drop in public support for democracy (Hetherington and Weiler 2009; Binder 2015; Lee 2015; Barber and McCarty 2016; Gerber and Schickler 2017; Svolik 2019). Yet while the polarization of US politics and society has been a prevalent phenomenon of American democracy since the 1970s, key questions about the scope, character, and implications of polarization in US foreign policy remain to be fully answered.

In recent years, the subject has begun to receive increased attention from international relations scholars and US foreign policy analysts (Walt 2019). One body of work has examined the extent to which polarization in US foreign policy is on the rise. The chief finding in these studies, which have focused mainly on congressional behavior or public attitudes, is that Democrats and Republicans have grown further apart on international issues, just as they have on domestic matters (DeLaet and Scott 2006; Kupchan and Trubowitz 2007; Snyder et al. 2009; Trubowitz and Mellow 2011; Peake et al. 2012; Gries 2014; Schultz 2017; Jeong and Quirk 2019; Smeltz et al. 2020). Related research shows how increased polarization is weakening the capacity of Congress to shape foreign policy or oversee the executive branch (Fowler 2015; Goldgeier and Saunders 2018**).** But other work has pointed to continuing areas of agreement or overlap between Democrats and Republicans, or ongoing congressional influence, on foreign policy (Chaudoin et al. 2010; Scott and Carter 2014; Busby et al. 2020; Tama 2020; Kertzer et al. 2021). Given these contrasting findings, more research is needed to fully understand the degree to which contemporary foreign policy debates are polarized, variation in political dynamics across areas of foreign policy, and the relationship between polarization and the behavior of policy making institutions.

Some scholars have also explored how foreign policy polarization can affect the outward-facing content or effectiveness of US foreign policy. The principal concern of this work is that polarization undermines bipartisan consensus for the grand strategy of liberal internationalism that often guided US foreign policy in the post-World War II era (Kupchan and Trubowitz 2007; Jervis et al. 2018). In the words of Peter Trubowitz and Peter Harris, hyper-partisanship, the absence of a compelling foreign policy narrative, and the erosion of the domestic social contract have weakened America’s “domestic political capacity to translate […] power assets into international influence” (Trubowitz and Harris 2019: 621). In a similar vein, Daniel Drezner writes that the “American foundations undergirding the liberal international order are in grave danger” (Drezner 2019: 10).^2^ Kenneth Schultz has highlighted additional effects of polarization, explaining how it makes US foreign policy less reliable, less capable of learning from past mistakes, and more vulnerable to harmful external influences (Schultz 2017). In a 2018 survey, US foreign policy professionals even ranked domestic polarization as the most critical threat to the USA (Smeltz et al. 2018). But some work points to the resilience of US support for liberal internationalism, highlighting ways in which domestic institutions and international realities constrain nationalist leaders from fully institutionalizing “America First” policies, and explaining why US leaders will continue to have incentives to pursue internationalist policies in an interconnected world (Chaudoin et al. 2018, 2021; Ikenberry 2020).

While these and other studies have provided a rich set of insights about the relationship between polarization and the content of US foreign policy, scholars have only begun to examine the impact of foreign policy polarization empirically. Moreover, the effects of polarization in particular foreign policy domains, such as international negotiations or military operations, are largely unexplored. With the existence and strength of a liberal international order being contingent in part on sustained US engagement, it is important to better understand the repercussions of polarization for America’s international commitment. The need for more research on these topics is only enhanced by the legacy left by Donald Trump, whose nationalist agenda and particularly partisan approach to politics presented especially strong challenges both to remaining reservoirs of bipartisanship and to key mechanisms of international cooperation (Jacobson 2017; Jervis et al. 2018; Stokes 2018).

With that context in mind, this special issue sets out to answer the following main questions: To what extent are US foreign policy debates polarized along partisan lines? How is polarization affecting the institutions of US foreign policy? And how is polarization changing the conduct or effectiveness of US foreign policy? In this overview article, we review new findings on these questions from articles in this special issue, situate those findings within prior scholarship on polarization and foreign policy, and suggest avenues for additional research that could further advance the frontier of knowledge in this area.

## Overall takeaways

The articles in this special issue present new research on the polarization of foreign policy ideas, the relationship between polarization and foreign policy institutions, and the effects of polarization on the conduct of US foreign policy. The articles cover an array of substantive issue areas, including military intervention, arms control, foreign policy spending, trade, and America’s international reputation. They also draw on many types of data, from conventional public opinion polls, to survey experiments, to information on a wide array of congressional activity. Moreover, the articles employ a variety of analytical methods, including statistical analyses, qualitative comparative analysis, and case studies. Collectively, the authors provide the most comprehensive look to date at the nature, extent, and implications of polarization on US foreign policymaking and execution.

The articles show how different types of polarization are manifested in contemporary US foreign policy, in both public attitudes and in the behavior of elected officials. On the extent of polarization, they illustrate the prevalence of ideological, social, and partisan divisions on foreign policy in recent years, but also suggest the need to incorporate some nuance into claims about polarization. Debates on issues ranging from climate change and immigration (Smeltz 2022), to arms control (Böller 2022), to the conduct of war (Lee 2022), to foreign policy spending (Bendix and Jeong 2022) reveal large and/or growing gaps between the preferences of Democrats or liberals, on the one hand, and Republicans or conservatives, on the other. Even views on some issues that previously exhibited little partisan divergence, such as attitudes toward China, Russia, and the Israel-Palestinian conflict, have recently become more polarized (Smeltz 2022). Members of Congress are also traveling abroad with lawmakers from the other party less often, reflecting a weakening social fabric on Capitol Hill (McGee and Theriault 2022).

At the same time, it remains surprisingly common for Democratic and Republican lawmakers to vote together on foreign policy (Bryan and Tama 2022), and presidents retain the capacity to achieve bipartisan support for certain kinds of military intervention (Maxey 2022). Moreover, on some issues, such as international trade and the scope of executive power, divisions within the parties or between Congress and the executive are at least as salient as divisions between liberals and conservatives (Bryan and Tama 2022; Friedrichs 2022; Homan and Lantis 2022). The upshot is that preference polarization is intensifying in many respects, but not uniformly and not to the exclusion of other political dynamics.

The articles also provide new insights and data on the effects of polarization on the institutions, execution, and effectiveness of US foreign policy. By making it more difficult for lawmakers to build the broad coalitions that are typically needed to enact laws, polarization is weakening congressional influence and enabling a further expansion in executive power, potentially facilitating rash or unwise presidential foreign policy actions (Marshall and Haney 2022). While lawmakers have sought to maintain congressional influence by shifting legislative activity to limitation riders—amendments to appropriations bills that restrict or prohibit certain types of spending—such devices are not well-suited to all types of foreign policy measures (Carcelli 2022).

Polarization also shapes and limits decisions on military action and international cooperation. Since Democrats place greater value than Republicans on avoiding civilian casualties and accord less importance than Republicans to achieving military victories, Democratic leaders tend to be more cautious with regard to the use of force than their Republican counterparts (Lee 2022). With regard to international cooperation, US presidents are becoming less capable of achieving Senate approval of international agreements as the prevailing ideology of Republican elected officials becomes more conservative (Böller 2022). At the same time, rising US preference polarization is weakening overseas confidence in America and reducing the willingness of citizens abroad to cooperate with the USA (Myrick 2022).

A few of the articles point to some more optimistic takeaways. The increased use by Congress of spending restrictions shows that lawmakers can adapt to political constraints in ways that enable them to maintain policy influence (Carcelli 2022). The role of ideology as a driver of congressional behavior can also enable Congress to act as a source of policy innovation (Bendix and Jeong 2022), while the intraparty divisions that characterize some foreign policy debates can foster the emergence of new ideas, policy entrepreneurs, and cross-cutting coalitions (Friedrichs 2022; Homan and Lantis 2022). Overall, though, the authors provide a variety of important cautionary tales about ways in which polarization can make it more difficult to carry out an effective foreign policy.

## Polarization and foreign policy ideas

A large literature has documented important differences between how liberals and conservatives view the world. Whereas liberals favor a cooperative approach to international politics and prefer non-military to military instruments of national power, conservatives are warier of multilateral mechanisms and more supportive of military might (Wittkopf 1990; Holsti 2004; Broz 2011; Rathbun 2012; Gries 2014; Milner and Tingley 2015; Jeong and Quirk 2019; Wenzelburger and Böller 2019; Raunio and Wagner 2020; Smeltz et al. 2020; Flynn and Fordham 2021). At the same time, US debates over some foreign policy issues, including international alliances, economic sanctions, humanitarian intervention, and human rights, do not break down consistently along left–right lines (Cutrone and Fordham 2010; Maxey, 2020; Busby et al. 2020; Tama 2020; Kertzer et al. 2021). In addition, many issues, such as international trade and foreign aid, involve both interparty and intraparty divisions (Thérien and Nöel 2000; Milner and Judkins 2004; Milner and Tingley 2010; Prather 2016; Rathbun 2016). In short, liberals and conservatives are strongly polarized on some major foreign policy questions, but the overall alignment between left–right ideology and foreign policy preferences is highly imperfect.

Prior work has also explored the relationship between public attitudes and the views of decision-makers or elites. On the one hand, scholars have established that citizens have certain core values that shape their worldviews, suggesting that the public forms some of their own judgments about international issues (Rathbun et al. 2016; Kertzer and Zeitzoff 2017). Public attitudes, in turn, sometimes influence the decision-making of leaders, particularly on salient issues (Aldrich et al. 2006; Foyle 2017). On the other hand, studies have shown that cues from leaders and other elites can themselves greatly shape public attitudes on foreign policy (Zaller 1992; Berinsky 2009; Guisinger and Saunders 2017). One consequence of this elite cue dynamic is that polarization among elites tends to filter down and exacerbate ideological polarization among the public (cf. Westwood 2019).

Given these interrelationships, it is important to understand how and to what extent foreign policy ideas are polarized among the public and decision-makers. Several of the articles in this special issue evaluate whether, to what extent, and how the views of citizens or leaders about US foreign policy are becoming polarized along left–right or other lines.

Dina Smeltz examines preference polarization in public attitudes on foreign policy, drawing on a long-running series of surveys conducted by the Chicago Council on Global Affairs (Smeltz 2022). She finds that polarization in public foreign policy views is increasing overall, but some issues are far more polarized than others. Democrats and Republicans still largely agree on the big-picture framework and goals of US foreign policy, supporting active engagement in the world, security alliances, overseas military bases, and international trade. Consistent with long-standing liberal-conservative divisions, polarization is much greater on some key means of foreign policy, such as the importance of multilateral institutions and military superiority. Most strikingly, polarization with respect to threat perceptions has jumped in recent years, with Republicans more concerned about hard security threats, such as the rise of China and international terrorism, and Democrats more concerned about the COVID-19 pandemic, climate change, and racial and economic inequality at home. These differences reveal that Democrats and Republicans do not even agree about which types of issues should demand government attention. Smeltz also discusses how presidential messaging on some hot-button issues, particularly under Donald Trump, has exacerbated polarization.

Sarah Maxey tackles another dimension of public attitudes, using original survey experiments to explore the influence of affective polarization on the American public’s support for military intervention (Maxey 2022). She finds that the impact of affective polarization on public support for military action varies across types of intervention and between Democrats and Republicans. Whereas affective polarization makes it harder for presidents to gain bipartisan support for interventions motivated by security goals, it does not limit the ability of presidents to achieve bipartisan backing for humanitarian interventions. In another important nuance, Democratic and Republican leaders face different political landscapes on the use of force, as the greater support of Republican citizens for military action makes it is easier for Democratic presidents than for Republican presidents to gain bipartisan support when deploying the military into combat. The upshot is that debates over the use of force are not immune to polarizing dynamics, but there exist important distinctions in the politics of military intervention debates.

William Bendix and Gyung-Ho Jeong consider the attitudes of elected officials, investigating the relative importance of ideological preferences and partisan calculations as drivers of congressional decision-making on amendments concerning defense and foreign aid spending (Bendix and Jeong 2022). Whereas much research on congressional decision-making focuses solely on the voting behavior of lawmakers, Bendix and Jeong evaluate contributors to both legislative cosponsorship and legislative votes. They find that ideological preferences strongly influence both cosponsorship and voting decisions, with liberals favoring amendments that limit defense spending and conservatives favoring amendments that limit foreign aid. While party identities also influence voting patterns on these limitation riders, ideological preferences play a more important role. More broadly, their study suggests that liberal and conservative ideologies greatly shape how members of Congress approach foreign policy issues.

Patrick Homan and Jeffrey Lantis offer a different take on congressional foreign policy views, highlighting the importance of establishment and anti-establishment factions within each party in debates over war powers (Homan and Lantis 2022). While most studies of congressional views hone in on the roles of liberal and conservative ideology, Homan and Lantis underscore differences in the attitudes of legislators regarding the exercise of power in Washington. Based on an examination of congressional votes concerning the use of force during the Obama and Trump administrations, they find that progressives and conservatives possessing anti-establishment views often split from their party leaders and align with each other in seeking to restrict the authority of the executive branch to deploy the military overseas. Even when such intraparty factions are relatively small, they have the potential to shape legislative outcomes when Congress is closely divided between the two parties, as it has typically been in recent years.

## Polarization and foreign policy institutions

The polarization of American politics and society severely challenges the democratic accountability of the political system and its agents, while changing how elected representatives carry out their roles and responsibilities (Sinclair 2016; Cayton and Dawkins 2020; Page and Gilens 2020). A key observation by scholars is that the alignment of political ideologies and partisanship has led to partisan conflict in Congress with the goal to prevent political achievements of the opposition (Theriault 2013; Hetherington and Rudolph 2015). Recent polling shows that the most salient reason why Americans identify with a political party—besides their party’s policies—is the conception that the other party will do harm to the country (Pew Research Center 2018). At the same time, partisan warfare has been exacerbated by the small size of congressional majorities and frequent turnover of party control on Capitol Hill in recent years, which provides parties with a greater incentive to focus on partisan competition (Lee 2016).

As a result of these trends, both parties have instituted procedural changes in order to increase their political success rate, including strengthening the party leadership to expedite partisan legislation and relying less on the seniority system when naming committee chairs (Cox and McCubbins 2005; Evans 2012; Wallner 2013; Smith 2014). The majority party has sought to suppress minority opposition to push through its policy agenda via the use of restricted and closed rules to prevent floor amendments to a bill as well as budget resolutions and reconciliation bills to avoid filibuster threats (Baumer and Gold 2010; Binder 2018). In contrast, the minority party increasingly relies on filibuster threats as well as on private actors and courts to implement legislative statutes (Binder and Maltzman 2009; Fukuyama 2014: 10ff.).

Partisan conflict has also become visible in the foreign policy realm, during the procedural and substantive foreign policy processes within Congress as well as in the relationship between Congress and the president (Auerswald 2006; Auerswald and Campbell 2012; Carter and Scott 2021). Some studies have shown a decline in the presidential use of international treaties due to the growing difficulty of marshaling bipartisan ratification majorities in Congress (DeLaet and Scott 2006; Peake et al. 2012; Buys 2017). Scholars have also demonstrated that congressional action on use of force questions is heavily shaped by partisan goals (Howell and Pevehouse 2007; Brulé 2008; Kriner 2010). Others find more generally that partisan conflict weakens the legislative branch’s role as check and balancer of the executive branch (Mann and Ornstein 2012; Fowler 2015; Franklin and Fix 2016; Goldgeier and Saunders 2018; Friedrichs 2021). Another body of work has shown how polarization has bred a new generation of congressional foreign policy entrepreneurs, who aim to change US foreign policy as well as their parties’ foreign policy position (Marsh and Lantis 2016; Homan and Lantis 2020). In response to congressional polarization, presidents increasingly circumvent a gridlocked legislative branch by exercising executive power unilaterally (see Rudalevige 2005; Hendrickson 2015; Edelson 2016; Burns, 2019). Partisan polarization also influences executive branch appointments, leading presidents to appoint fewer officials from the opposition party to senior positions (Flynn 2014).

Several articles in the special issue take a closer look at changes to the institutions involved in US foreign policymaking due to polarization. Combined, they add to our understanding of Congress’ role and presidential power in contemporary US foreign policymaking.

Bryan Marshall and Patrick Haney assess changes to the role of Congress in the foreign policy realm against the background of public expectations for strong US global leadership (Marshall and Haney 2022). Through analyses of congressional behavior such as voting, lawmaking, and oversight, they show that partisan polarization has led to a decline in congressional power in foreign affairs. They find evidence of partisan incentives for congressional abdication to the executive branch in a decline in the number of foreign policy laws enacted by Congress, fewer checks on presidential positions by congressional foreign policy committees, a weakening of Congress’ power of the purse, and a growing tendency for presidential unilateralism. Overall, Marshall and Haney warn that these developments have created a serious imbalance in the institutional authority of Congress and the president to execute US foreign policy, and that this imbalance has the potential to jeopardize American democracy.

James Bryan and Jordan Tama examine the frequency and character of congressional bipartisanship on international issues compared to domestic ones (Bryan and Tama 2022). Analyzing an original data set of nearly 3000 important congressional votes since the end of the Cold War, Bryan and Tama find that severe cases of polarization, in which more than 90 percent of the members of the two parties vote on opposite sides, remain the exception in US foreign policy debates. In addition, they find that, overall, Congress is less polarized on international issues than on domestic issues. By distinguishing among different forms of foreign policy bipartisanship—pro-presidential, anti-presidential, and cross-partisanship—they provide a more nuanced conception of the relationship between partisan polarization and bipartisanship rates in Congress. While pro-presidential bipartisanship characterizes most foreign policy votes, anti-presidential bipartisanship occurs strikingly often as well. Moreover, Bryan and Tama find that bipartisanship rates vary across policy domains, with international security issues enjoying more bipartisan consensus than international economic issues. Overall, their findings suggest that despite increasing ideological distance between members of both parties, Congress remains capable of addressing international issues and enacting its constitutional role as check and balancer of the executive branch.

Shannon Carcelli argues that polarization and gridlock in Congress have incentivized legislators to turn to unorthodox foreign policymaking, particularly on foreign aid policy (Carcelli 2022). Through the use of limitation riders (specifications written within an appropriations bill that limit the administration’s policy discretion), members of Congress seek to limit the allocation of aid to other countries or prevent spending foreign aid money for particular purposes. Carcelli shows that through the shift from more traditional means of foreign policy legislating to the increased use of appropriations bills to shape foreign policy, Congress has been quite assertive and influential despite high levels of partisan polarization. Quantitatively, she finds evidence for an increase in the use of foreign aid limitation riders as polarization increases. Qualitatively, she shows how the content of limitation riders is stronger in high-polarization eras, as the topics of riders shift from budgets and contracting issues to more substantive specifications regarding recipient countries and types of aid. In addition, her findings indicate that an increase in congressional conservatism decreases the use of limitation riders, suggesting that liberal majorities struggle more to authorize foreign aid by traditional means.

Zachary McGee and Sean Theriault investigate the causes and consequences of partisanship in congressional travels abroad (McGee and Theriault 2022). They observe that congressional travels abroad have historically facilitated cordiality and civility in Congress, as well as motivated legislators to reach across the aisle to foster bipartisan consensus. Their analysis of overseas trips, known as Congressional Delegations (CODELS), from 1977 to 2018 reveals that Democrats with ideologically extreme views travel abroad more often only with co-partisans, and that both Democrats and Republicans with ideologically extreme views engage in fewer bipartisan trips abroad. Combined, their results suggest that ideological extremism and partisan warfare are shaping not only the voting behavior, but also the social interactions, of members of Congress. This increased social polarization, in a vicious cycle, threatens in turn to exacerbate polarization in the legislative process: just as party polarization decreases the opportunity for members of Congress to develop personal relationships with each other, the lack of strong personal relationships across the aisle makes it more difficult for members of Congress to craft bipartisan legislation later.

## Polarization and foreign policy effectiveness

Domestic polarization not only affects the policymaking process but also the way policies become implemented. Scholars of US foreign policy have shown that over time, the highly contentious forces of domestic politics in some policy areas, such as international economics, have incentivized the president to rely more heavily on other instruments, such as military deployments and defense procurement (Milner and Tingley 2015). Others have shown that partisan incentives have reduced presidents’ use of force abroad and influenced how presidents carry out military operations (Howell and Pevehouse, 2007; Kriner 2010). Meanwhile, others have found that greater prospects for legislative success incline the president to engage in more high-risk military interventions and fewer humanitarian interventions (Marshall and Prins 2016). In addition, studies on presidential use of executive agreements suggest that domestic polarization inclines presidents to advance their agenda unilaterally (Caruson and Farrar-Myers 2007; Amirfar and Singh 2018).

Polarization also influences the effectiveness of US foreign policy execution. As some scholars have argued, polarization has limited the rally-around-the-flag effect in response to external security threats (Myrick 2021). In addition, political and material costs for the execution of certain partisan foreign policies and visions of international order have increased in times of domestic polarization (Bafumi and Parent 2012; Kreps et al. 2018). As a consequence of the widening gap domestically for a cohesive strategy, the executive branch is more incentivized to politicize US foreign policy through wedge-issues to weaken political opposition (Snyder et al. 2009). This, in turn, has contributed to a polarization of US relationships with allies and partners (Drezner 2018).

Several articles in the special issue trace the consequences of domestic polarization for the conduct and effectiveness of US foreign policy. These articles tackle the larger question of whether and how polarization undermines America’s global power and standing across a range of issue areas.

Florian Böller analyzes the conditions under which the US role in arms control policy has shifted from a “booster” that initiates new agreements, to a “brakeman” that restricts or puts a halt to them (Böller 2022). Arms control treaties have been a key element of the liberal international order under US global leadership and have contributed to global security and disarmament. Via a crisp-set Qualitative Comparative Analysis, he evaluates 24 cases of US decisions on international arms control treaties from 1963 to 2021, followed by a brief case study of the Trump administration’s withdrawal from several agreements. Böller finds that the strength of conservative treaty skeptics in the US Senate contributed to the demise of arms control policies since the end of the Cold War. In turn, the president’s party affiliation appears to be less decisive for the US role, although Democratic presidents are more likely to support a booster role. In addition, the perceived reciprocity, or “tit for tat,” embedded in the treaty, is an important condition that shapes US treaty behavior. Overall, the ideology of senators, particularly their skepticism toward multilateral commitments, places a key structural constraint on US international treaty making. As a result, Böller argues, changes in the presidency, from Trump to Biden, for example, are insufficient to transform US arms control policy.

Gordon Friedrichs examines the extent and impact of intraparty polarization in Congress on US trade policy (Friedrichs 2022). Facilitating trade liberalization through multilateral organizations and free trade agreements has been characteristic of US global leadership and the liberal international order since the end of World War II. By relying on a new dataset of congressional letters and cosponsorship alliances, Friedrichs derives trade policy preferences from members of Congress and their ideological dispositions. Via a structured-focused comparison of two cases of US trade policy—the Trans-Pacific Partnership Agreement and the US-Mexico-Canada-Agreement—he shows that both parties are intrinsically polarized between free and fair trade preferences. However, the relative strength of ideological factions within both parties determines when legislators can build cross-cutting coalitions in support of trade agreements. In addition, both case studies reveal that intraparty preference polarization incentivizes the executive branch to pursue side-payments and issue linkage in international trade negotiations, in an effort to forge cross-party ratification majorities. Overall, these findings indicate that interparty polarization induces intraparty polarization and bears the potential to generate foreign policy realignments.

Carrie Lee investigates how polarization impacts presidents’ decisions to use force abroad (Lee 2022). Lee argues that liberals and conservatives interpret wartime casualties very differently from each other, which has significant implications for public support for war and presidential decisions regarding military intervention. Using three survey experiments, she assesses how ideology and party influence casualty sensitivity. Her findings reveal that conservatives are less likely than liberals to change their support for military operations in response to increases in casualties. This pattern holds under both Democratic and Republican presidents, suggesting that ideology rather than partisanship is the key driver of it. Lee’s results suggest that Republican presidents have strong incentives to pursue gains on the battlefield, while Democratic presidents have strong incentives to limit the number of US casualties. With polarization steadily increasing, ideological differences in the public’s perception of warfare will likely translate into wide swings in military strategy from administration to administration, undermining international trust in US security leadership.

Rachel Myrick examines how partisan polarization affects British perceptions of US security commitments and global leadership (Myrick 2022). The US–UK special relationship has been considered a pillar of the liberal international order and one that has proven particularly resilient despite various crises. Based on a survey experiment involving 2000 adults in the United Kingdom, Myrick demonstrates how US domestic polarization negatively impacts British citizens’ perception of the bilateral relationship. She shows how foreign perceptions of extreme US polarization weaken British willingness to engage in future partnerships with the US because of an erosion of a bipartisan commitment to liberal norms, multilateralism, and an open world economy. Furthermore, her findings reveal that polarization in the US increases skepticism of American global leadership due to anticipation that US foreign policy will be more likely to change with executive turnover and thus be less predictable. She further highlights that these negative reputational consequences due to polarization are primarily driven by preference polarization between Republicans and Democrats, rather than by affective polarization. Myrick’s findings contribute to our understanding of some of the broader international consequences of domestic polarization, particularly in increasing uncertainty around future US foreign policy.

## Avenues for further research

Collectively, the articles in this special issue underscore both the progress that has been made in the study of polarization and US foreign policy and the fertile ground that exists for further research in this area. The answers to some questions related to polarization and foreign policy are clear from these and other studies. On the whole, foreign policy has not been immune from the rising levels of preference, affective, social, and partisan polarization that have characterized American politics in recent decades. From the public to members of Congress, Democrats and Republicans have diverged in their views and behavior on international issues. This rising polarization has had a series of concerning effects, from an increase in US unilateralism to a decline in America’s international cooperation capacity and overseas reputation. At a time when a variety of policy challenges call out for enhanced international engagement and cooperation, polarization is making it harder for the US to contribute constructively to global problem-solving and international order maintenance.

Yet some key debates about polarization and foreign policy remain unresolved. While it is clear that the foreign policy views and behavior of Democrats and Republicans have diverged, scholars differ in their assessment of the extent to which members of each party have become ideologically homogenous. While some of the studies in the special issue highlight the separation of Democrats and Republicans into ideologically distinct camps on foreign policy (Bendix and Jeong 2022; Böller 2022), others highlight persistent intraparty divisions on international issues (Friedrichs 2022; Homan and Lantis 2022). More research is needed to further enhance understanding of the relative importance of interparty polarization and intraparty divisions. Further studies could also shed light on the specific alignment of different types of polarization (ideological, affective, social, partisan) across and within foreign policy areas. Given existing comprehensive work on the causes and drivers of polarization in the domestic context, additional work on the extent to which the repercussions of US global interdependencies and costly public goods provisions have become themselves drivers of partisan or ideological polarization could be particularly promising (cf. Colgan and Keohane 2017; Norrlöf 2018). Such work should be particularly timely in the wake of Donald Trump’s presidency considering the ways in which some of his foreign policy positions—for instance, on trade and the US overseas military presence—diverged from Republican orthodoxy.

Further research is also needed to bolster understanding of the extent to which Congress is still able to constrain the president in this highly polarized era. Overall, articles in the special issue show that polarization is weakening Congress on foreign policy, in areas ranging from legislating, to oversight, to overseas travel (Haney and Marshall 2022; McGee and Theriault 2022). Yet Congress continues to challenge or restrain the president on some important international issues, suggesting that there remain substantial limits on presidential power (Bryan and Tama 2022; Carcelli 2022). One way to move this debate forward would be to trace the motivations of legislators in seeking to manipulate the foreign policymaking process—for instance, examining when legislators act to advance their own policy views and when they act based on partisan goals. This would enhance understanding of the conditions that facilitate bipartisan rather than partisan congressional opposition to presidential policies. Another way to advance this debate would be to shift some attention from congressional behavior to presidential decision-making, using process-tracing or other qualitative methods to examine the extent to which the president and other executive branch officials take into account congressional views and positions when making important foreign policy decisions.

New studies could also further investigate the effects of US polarization on the behavior of other countries and the stability of international order. To date, most work on the foreign policy effects of polarization, including several of the special issue papers, has focused on how polarization can alter policy making dynamics in Washington or change the international conduct of the USA (Schultz 2017; Böller 2022; Friedrichs 2022; Lee 2022). This body of work presents important findings on the reliability of American democracy and US capacity to provide global public goods in the light of domestic division, populism, and institutional corrosion (cf. Daalder and Lindsay 2018; Diamond 2019; Musgrave 2019; Norrlöf 2020). Myrick takes this research agenda a step further by investigating the effects of polarization on overseas attitudes toward the USA (Myrick 2022). An implication of her paper is that if overseas attitudes change, the behavior of foreign governments will also likely change. But to what extent are foreign governments actually acting differently—for instance, by pursuing alternatives to partnerships with the US—based on concern that they can no longer rely on US leadership or commitments? Moreover, to what extent and when do US adversaries try to take advantage of weaknesses of US foreign policy caused by domestic polarization? More broadly, to what extent is increased domestic polarization contributing to changes in the formal and informal architecture of the international order? These may be among the most important and fruitful avenues for new research and they are particularly relevant against the background of established insights that democracies are reliable cooperation partners and formidable in military warfare (Lake 1992; Bueno de Mesquita et al. 1999; Doyle 2005).

Finally, it would be worthwhile for scholars of foreign policy to build more connections between research on US polarization and work on polarization in other political contexts. To a large extent, scholarship on the politics of US foreign policy has been conducted in isolation from scholarship on the politics of foreign policy in other countries (cf. Somer & McCoy 2018; Carothers 2019). But some recent work highlights important commonalities in party politics on foreign policy across contemporary democracies, underscoring the value of integrating knowledge about the US case with knowledge about other cases (Wagner et al. 2017; Oktay 2018; Haesebrouck and Mello 2020; Raunio and Wagner 2020; Wagner 2020; see also Beasley et al. 2013). While some characteristics of American politics, such as its two-party system, presidential democracy, electoral college system, and tradition of exceptionalism, mean that neither polarization dynamics nor the content of foreign policy will ever be identical in the US and other democracies, we should strive to enhance our understanding of similarities and differences in the extent, drivers, and effects of polarization in countries across the globe.

## Footnotes

1 This definition draws on Lee 2015, 263.

2 Other scholars highlight limits in the extent of bipartisan foreign policy consensus or in the scope of the liberal international order even during the Cold War (Rathbun 2012, Acharya 2018, Allison 2018; see also McCormick and Wittkofpf 1990; Meernik 1993).
